# Exposure to phenanthrene affects oocyte meiosis by inducing mitochondrial dysfunction and endoplasmic reticulum stress

**DOI:** 10.1111/cpr.13335

**Published:** 2022-09-20

**Authors:** Yi Wang, Si‐Hong Li, Shu‐Jie Yang, Xiao‐Qing Li, Lu Liu, Xiang Ma, Dong Niu, Xing Duan

**Affiliations:** ^1^ Key Laboratory of Applied Technology on Green‐Eco‐Healthy Animal Husbandry of Zhejiang Province, Zhejiang Provincial Engineering Laboratory for Animal Health Inspection & Internet Technology, Zhejiang International Science and Technology Cooperation Base for Veterinary Medicine and Health Management, China‐Australia Joint Laboratory for Animal Health Big Data Analytics College of Animal Science and Technology & College of Veterinary Medicine, Zhejiang A&F University Hangzhou China

## Abstract

**Objectives:**

Phenanthrene (PHE) is one of the most abundant polycyclic aromatic hydrocarbons (PAHs), which is a widespread environmental contaminant. Various studies showed that PHE has adverse impacts on animals and human health. It has been shown that PHE exposure induced follicular atresia and endocrine dyscrasia in female mice. However, the potential mechanism regarding how PHE affects female reproductive system especially the oocyte quality has not been elucidated.

**Methods and Results:**

In this study, we set up PHE exposure model and found that PHE exposure compromised oocytes maturation competence by inhibiting spindle assembly and chromosomes alignment. Moreover, PHE exposure induced mitochondrial dysfunction and endoplasmic reticulum (ER) stress, leading to increased reactive oxygen species (ROS) and aberrant calcium levels in cytoplasm, eventually induced oxidative stress and DNA damage in oocytes. Furthermore, we found that oral administration of PHE caused the occurrence of oxidative stress and apoptosis in female ovary. In addition, the oocyte exhibited aberrant spindle morphology and failure of actin cap formation in metaphase II oocytes.

**Conclusions:**

Taken together, our study demonstrated that mitochondrial dysfunction and ER stress‐induced oxidative stress and DNA damage are the major cause of poor oocyte quality after PHE exposure.

## Graphical abstract

Mechanism of abnormal meiotic progression caused by PHE exposure. The current study indicated that PHE exposure caused ER stress and mitochondrial dysfunction by disrupting intracellular redox and Ca^2+^ homeostasis, eventually inducing DNA damage and aberrant oocyte maturation.

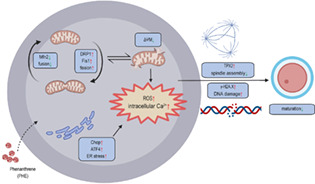

## 1 INTRODUCTION

Polycyclic aromatic hydrocarbons (PAHs), are a group of environmental pollutants that widely found in environment.^1^, ^2^ PAHs are produced by the incomplete combustion of organic material and released into the environment, such as industrial processes, waste incineration, traffic emissions, tobacco and coal burning. Because of their low solubility and lipophilic in nature, PAHs can easily accumulate in human body through oral, dermal and inhalation routes.^2^, ^3^, ^4^, ^5^, ^6^ It has been demonstrated that PAHs are endocrine disruptors and have toxic effects on diverse organisms, especially on reproductive system. More recent study indicated PAHs exposure are closely associated with reproductive disorders, including infertility, premature birth and low birth weight.^7^ Moreover, PAHs have been found in human serum and were positively correlated with the abnormal sex hormone secretion, the occurrence of polycystic ovary syndrome and premature ovarian failure in women.^8^ Meanwhile, the toxic effects of PAHs could transmit to subsequent generations.^9^ Therefore, the adverse effect of PAHs on the human has drawn more considerable attention.^3^, ^10^, ^11^, ^12^, ^13^

Phenanthrene (PHE), a type of low‐molecular‐weight PAH, was widely distributed in environment.^14^ Several studies have reported that the concentration of PHE was the highest of the 16 PAHs compounds in drinking water and air.^15^, ^16^ As with other PAHs, long‐term exposure to PHE has negative effects on various systems in organisms. Orally and intraperitoneal injection of PHE in rats induced the occurrence of oxidative stress and inflammation in liver and lung.^15^ Moreover, PHE could induce hepatic lipid metabolism disorder via changing the composition of gastrointestinal bacteria in mice.^17^ In addition, long‐term exposure to environmental levels of PHE reduced the number of sperm and Sertoli cells. PHE exposure increased the level of apoptosis in testicle cells and altered the expression levels of oestrogen receptor, androgen receptor and aromatic hydrocarbon receptor in male mice.^18^ It has been shown that PHE exposure increased follicular atresia and inhibited ovarian development by disrupting the function of hypothalamus–pituitary–gonadal (HPG) axis and the steroidogenic pathway aquatic organisms.^19^, ^20^, ^21^ In addition, gestational exposure to PHE caused reproductive disorders in F1 adult female mice, which exhibited by the increased number of antral follicles and reduced levels of luteinizing hormone (LH) and estradiol in serum.^22^ Although the toxic effect of PHE on ovarian function and sex hormone secretion has been demonstrated, the effect and potential molecular mechanisms of PHE‐induced deterioration of oocyte quality remain unclear.

Oocytes are arrested in the prophase of meiosis I and resume meiotic maturation in response to luteinizing hormone surge. After germinal vesicle breakdown (GVBD), spindle is organized at or near centre of oocyte, which subsequently migrates to oocyte cortex. Shortly after, polar body extrusion occurs, which is accompanied by segregation of homologous chromosomes. Therefore, oocyte maturation is a perquisite for successful fertilization and embryonic development.^23^ Aberrant microtubules assembly and chromosomes segregation cause aneuploidy and spontaneous abortion in mammals.^24^, ^25^ The normal distribution and function of organelles, such as mitochondria and endoplasmic reticulum (ER), are essential for oocyte maturation. Mitochondria not only regulates the energy metabolism, but also maintains the homeostasis of intracellular Ca^2+^. Increase of cytosolic Ca^2+^ concentration disrupts oxidative phosphorylation and redox homeostasis, or opens the mitochondrial permeability transition pore, which further compromises mitochondrial function.^26^ Mitochondrial dysfunction usually causes excessive accumulation of reactive oxygen species (ROS), and further induces the occurrence of oxidative stress, DNA damage and impairs developmental competence of oocytes.^23^, ^27^, ^28^ ER is an important organelle that responsible for folding and assembly of secretory proteins. Accumulation of misfolded or unfolded proteins in ER can disrupt ER homeostasis and active ER stress. Activation of ER stress can trigger unfolded protein response (UPR), which further deteriorates oocyte quality.^29^

In the present study, we determined the effect of PHE on oocyte quality by setting up in vitro and in vivo PHE exposure model. Our results showed that PHE exposure caused failure of oocyte maturation in vitro through inducing mitochondrial dysfunction, oxidative stress and ER stress, and further caused the occurrence of DNA damage. Moreover, by constructing PHE‐exposed in vivo mice model, we found that PHE exposure induced occurrence of oxidative stress and apoptosis in ovary. Additionally, the quality of MII oocytes‐derived from in vivo were reduced which exhibited by the aberrant cytoskeleton assembly. These data will expand our knowledge of how PHE deteriorates oocyte quality and ovarian function in mammals.

## 2 MATERIALS AND METHODS

### 2.1 Animals and feeding regimens

All mice were housed at controlled condition temperature (25 ± 2°C) and in 12:12‐h light/dark cycle. Water and food were provided and free access throughout the study. All procedures were in accordance with the guidelines of the Institutional Animal Care and Use Committee at Zhejiang A&F University, China.

Thirty mice (body weight: 28–30 g/mice) were randomly divided into two groups. The mice were continuously oral gavage administration of PHE (dissolved in corn oil) at dose of 0 and 5 mg/kg/day^17^ for 30 consecutive days.

### 2.2 Oocyte collection and culture

Germinal vesicle (GV) oocyte collection and culture: 6–8‐week‐old ICR female mice were intraperitoneal injection of 10 IU of pregnant mare serum gonadotropin (PMSG, dissolved in PBS; 100 μl per mice); after 48 h, cumulus‐oocyte complexes (COCs) were isolated from ovaries in M 2 medium, then clear the cumulus cells away from COCs by repeated mouth‐controlled pipetting. The oocytes were then cultured with M 16 medium (Sigma Aldrich) under mineral oil at 37°C in 5% CO_2_.

PHE was dissolved in DMSO to prepare a 50 mM stock solution and then diluted in M 16 medium at different concentrations for experiments. Based on the effect of PHE on GVBD and first polar body extrusion of MI oocytes, 400 μM was chose for the following experiments.

Metaphase II (MII) collection and culture: 5 IU human chorionic gonadotrophin (HCG) was injected at 48 hours after PMSG injection. After 13–14 h, female mice were super‐ovulated, and MII oocytes were collected from ampulla of the oviduct.

### 2.3 Immunofluorescence and confocal microscopy

For immunofluorescent staining, oocytes were fixed in 4% paraformaldehyde in PBS at room temperature for 30 min and then transferred to membrane permeabilization solution (0.5% Triton X‐100) for 20 min. The oocytes were blocked with 1% bovine serum albumin in PBS for 1 h and incubated at 4°C overnight with primary antibody at the concentration indicated in the instructions, specifically, mouse monoclonal anti‐a‐tubulin‐FITC antibody and phalloidin‐TRITC were purchased from Sigma (St. Louis, MO), rabbit polyclonal anti‐Drp1, rabbit polyclonal anti‐γ‐H2AX antibody was purchased from Cell Signaling Technology (Danvers, MA), rabbit polyclonal TPX2 antibody was purchased from Novus Biologicals (Colorado, USA). After washing with 0.1% Tween 20 and 0.01% Triton‐X 100 for 3 times (10 min/time), the oocytes were incubated with secondary antibody for 1 h at room temperature. Finally, the oocytes were stained with Hoechst 33,342 for 10 min. The samples were mounted on glass slides and observed under confocal laser‐scanning microscopy (FV3000, Olympus).

### 2.4 Mitochondrial membrane potential, ER distribution, intracellular calcium and ROS level assay in MI oocytes

The mitochondrial membrane potential, ER distribution, intracellular calcium and ROS level were determined by using MitoProbe JC‐1, ER‐tracker red kit, Fluo 4‐AM probe and ROS Assay Kit according to the manufacturer's instructions (Beyotime, China). Briefly, the oocytes were incubated with probes in suitable concentration for 30 min at 37°C, after three times of washing with fresh M 16, oocytes were subjected to confocal microscopy imaging.

### 2.5 Real‐time quantitative PCR

Gene expression was determined by real‐time quantitative PCR and calculated using the 2^−ΔΔCT^ method. Total RNA was extracted from 50 oocytes using Dynabeads mRNA DIRECT Purification Kit (Invitrogen). The first strand cDNA was prepared using HiScript II 1st Strand cDNA Synthesis Kit (Vazyme) and quantitative PCR (qPCR) was conducted using the Hieff qPCR SYBR Green Master Mix (Yeasen).

### 2.6 Quantification of immunofluorescence imaging

After immunofluorescent staining, the control and treated oocytes samples were mounted on glass slides and observed under confocal laser‐scanning microscopy using the same parameters. The average fluorescence intensity within the region of interest (ROI) of immunofluorescence images was examined by the Fiji software. The Wolfram Mathematica software was used to curve the fluorescence intensity plotting of cortical actin and standardize the result.

### 2.7 Statistical analysis

At least three independent biological replicates were examined for analysis. The statistical analysis was performed with the GraphPad Prism software 8.0.1 (La Jolla, USA). All the analysis of data was performed by one‐way analysis of variance with Turkey's test for multiple comparisons.

## 3 RESULTS

### 3.1 Effects of PHE exposure on oocyte maturation in vitro

**FIGURE 1:**
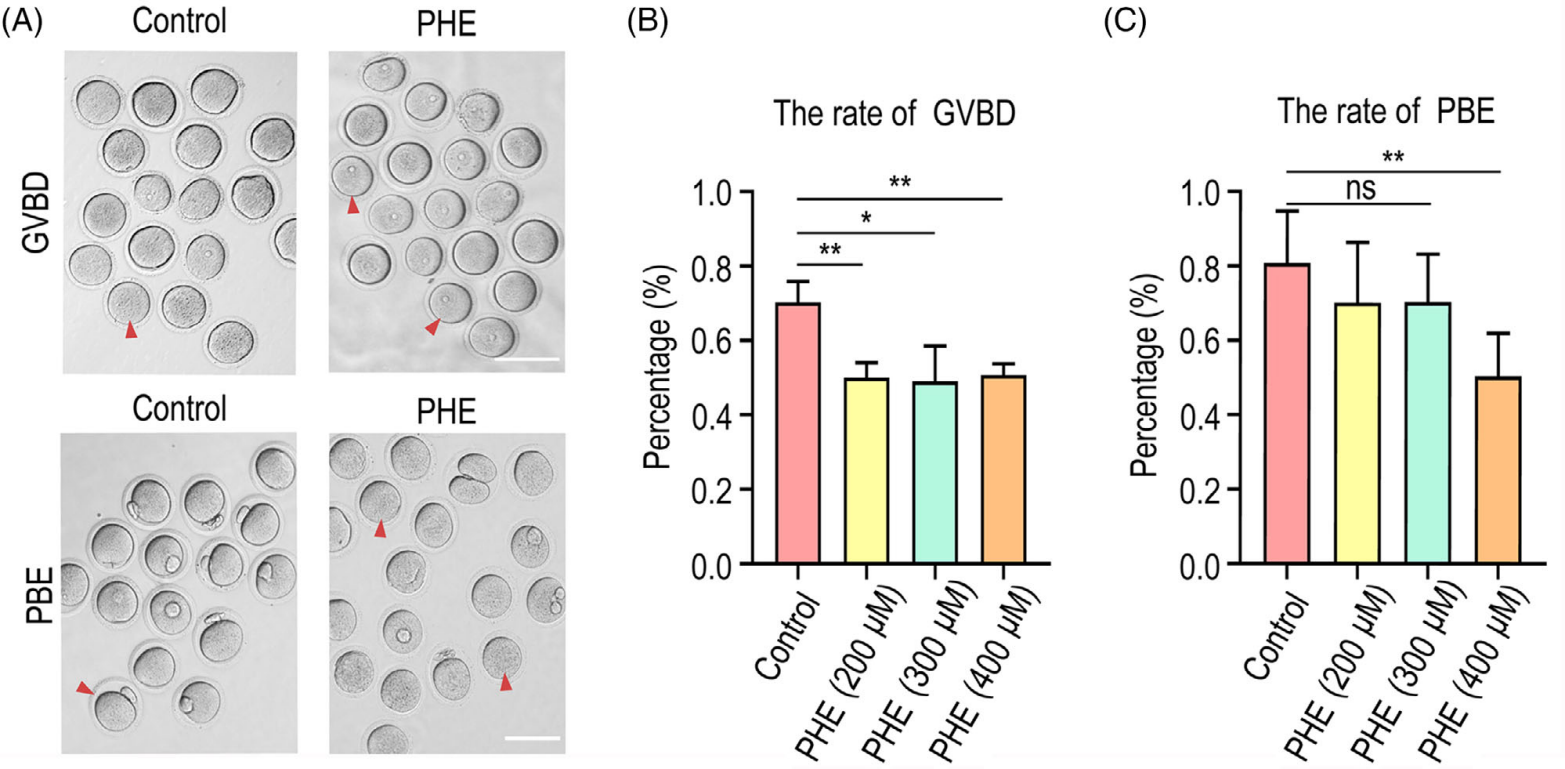
Phenanthrene (PHE) exposure impairs oocyte maturation. (A) Representative images of germinal vesicle breakdown and polar body extrusion oocytes in control and PHE‐exposed oocytes (arrowheads: the oocytes underwent germinal vesicle breakdown (GVBD) and polar body extrusion in control group, but failed GVBD and polar body extrusion in PHE‐treated group). Scale bar, 100 μm. (B) Percentage of GVBD was quantified in control and oocytes exposed to different concentrations of PHE (200, 300 and 400 μM). (C) Representative images of first polar body extruded‐oocytes from control and PHA‐exposed groups. Scale bar, 100 μm. Data are represented as mean ± SD from at least three independent experiments. ns *p* > 0.05, *p < 0.05 and **p < 0.01

To investigate the toxic effects of PHE on oocytes maturation, the rates of GVBD and first polar body extrusion were examined after exposure to different concentrations of PHE in vitro. After exposure to PHE, the percentage of GVBD (Figure 1A,B) and the first polar body extrusion (Figure 1A,C) significantly reduced in a dose‐dependent manner, indicating that PHE exposure compromised meiotic maturation in female mouse oocytes. Since the differences of GVBD and first polar body extrusion rates were the most pronounced at 400 μM (GVBD: 70.3% ± 5.5%, *n* = 100 versus 50.7% ± 3.1%, *n* = 150, *p <* 0.01, first polar body extrusion rates: 80.8% ± 14.0%, *n* = 100 versus 50.4% ± 11.5%, *n* = 150, *p <* 0.01), we performed the following experiments using 400 μM of PHE.

### 3.2 PHE disturbs MI spindle assembly and causes chromosome misalignment during oocyte meiotic maturation

**FIGURE 2:**
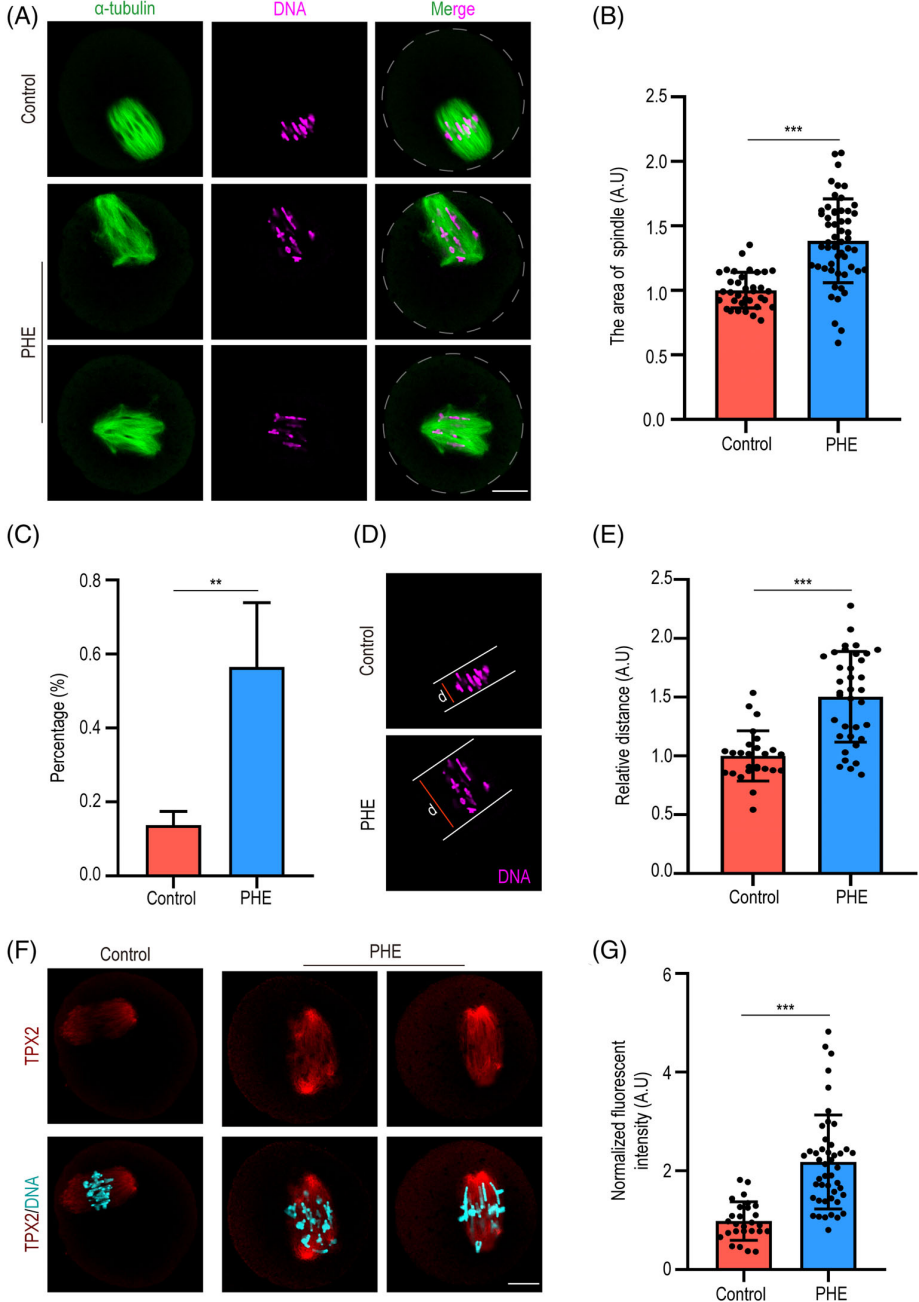
Phenanthrene (PHE) exposure disturbs MI spindle assembly and chromosome alignment. (A) Representative images of spindle morphologies in control and PHE‐treated group. Scale bar, 20 μm. (B) Spindle area was quantified in control and PHE‐exposed oocytes. (C) The percentage of aberrant spindle morphology was quantified after PHE treatment. (D,E) Quantification of chromosomes distance in control and PHE‐exposed oocytes. (F) Representative images of TPX2 in control and PHE‐treated groups. Scale bar, 20 μm. (G) Normalized fluorescent intensity of TPX2 in control and PHE‐treated groups. Each point in the histogram represents the number of oocytes. Data are represented as mean ± SD from at least three independent experiments. **p < 0.01, ***p < 0.001

To evaluate the reason for defect in oocyte meiosis after exposure to PHE, we determined spindle assembly and chromosome alignment during oocyte maturation. As shown in Figure 2A–C, we observed that the area of spindles in treatment group was significantly higher than controls (1.0 ± 0.1, *n* = 35 versus 1.4 ± 0.3, *n* = 54, *p* < 0.001). Importantly, the control group oocyte presented a typical barrel‐shaped spindle. Whereas the PHE‐treated oocytes showed multipolar spindles, and quantitative results showed that the percentage of aberrant spindle morphology was significantly higher in PHE‐treated group than control group (13.8% ± 3.7%, *n* = 150 versus 56.5% ± 17.4%, *n* = 100, *p <* 0.01). During anaphase of oocyte meiosis, chromosomes were aligned neatly on the spindle equator by microtubules to avoid aneuploidy. Our results showed that most of control oocytes exhibited a well‐aligned chromosome on the equatorial plate. In contrast, the occurrence of chromosomes misalignment in PHE‐treated group was obviously increased. To further quantitatively evaluate the misalignment of chromosomes, we measured the relative spread distance of chromosomes and found that the spread distance of chromosomes was significantly increased after PHE treatment (1.0 ± 0.2, *n* = 26 versus 1.5 ± 0.4, *n* = 36, *p <* 0.001; Figure 2D,E). Interestingly, we found that TPX2 (Targeting Protein for Xklp2), which is an important protein in microtubule nucleation and stability, was significantly increased after PHE treatment (1.0 ± 0.4, *n* = 27 versus 2.2 ± 0.9, *n* = 46, *p <* 0.001; Figure 2F,G). Thus, our results suggested that the aberrant expression of TPX2 might be associated with aberrant microtubule nucleation and chromosomes misalignment after PHE exposure.

### 3.3 PHE disturbs mitochondrial function in oocytes

**FIGURE 3:**
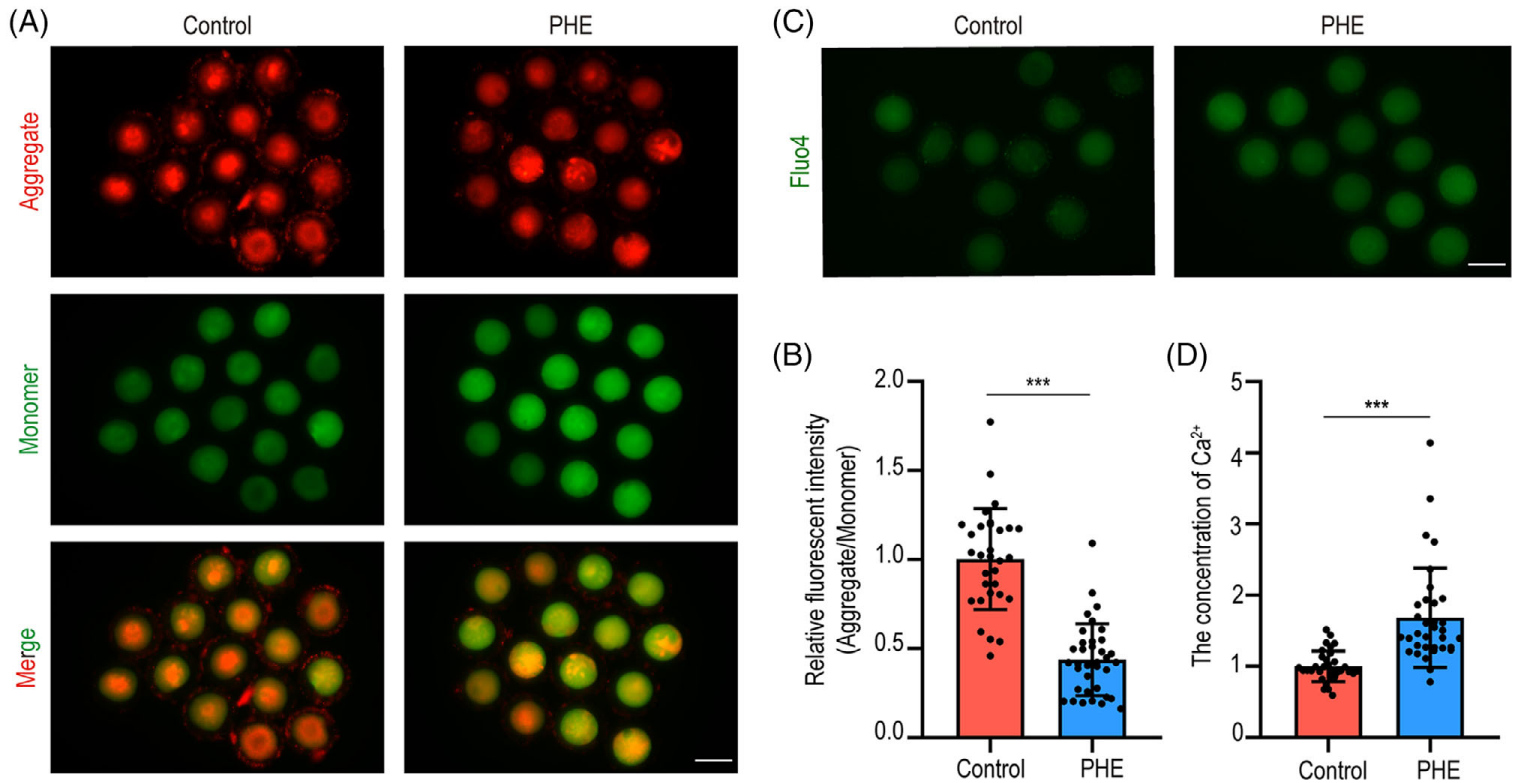
PHE exposure affects mitochondrial function in oocytes. (A) Representative images of JC‐1 kit staining in control and PHE‐exposed oocytes. Scale bar, 100 μm. (B) Mitochondrial membrane potential was recorded after PHE‐exposed (Aggregate/Monomer). (C) Representative images of cytosolic Ca^2+^ levels in oocytes after PHE treatment. Scale bar, 100 μm. (D) Fluorescent intensity of Ca^2+^ levels were quantified in control and PHE‐treated groups. Each point in the histogram represents the number of oocytes. Data are represented as mean ± SD from at least three independent experiments. ***p < 0.001

To further examine the potential toxic mechanism of PHE in oocyte, the mitochondrial membrane potential was assessed by JC‐1 staining, which was an important criterion for evaluating mitochondrial function and activity. We found that PHE treatment led to an increase in monomers and a reduction in aggregate in oocytes (Figure 3A). The quantitative results, the fluorescent intensity of the aggregate/monomer, showed that the mitochondrial membrane potential in PHE treatment group was lower than controls (1.0 ± 0.3, *n* = 31 versus 0.4 ± 2.0, *p <* 0.001; Figure 3B), indicating that PHE has negative influence on mitochondrial function. Mitochondrial dysfunction was often accompanied by calcium homeostasis imbalance. As expected, our results showed that the intracellular Ca^2+^ levels were significantly increased after PHE treatment (1.0 ± 0.2, *n* = 32 versus 1.7 ± 0.7, *n* = 34, *p <* 0.001; Figure 3C,D).

### 3.4 PHE perturbs mitochondrial dynamics and leads to ER stress

**FIGURE 4:**
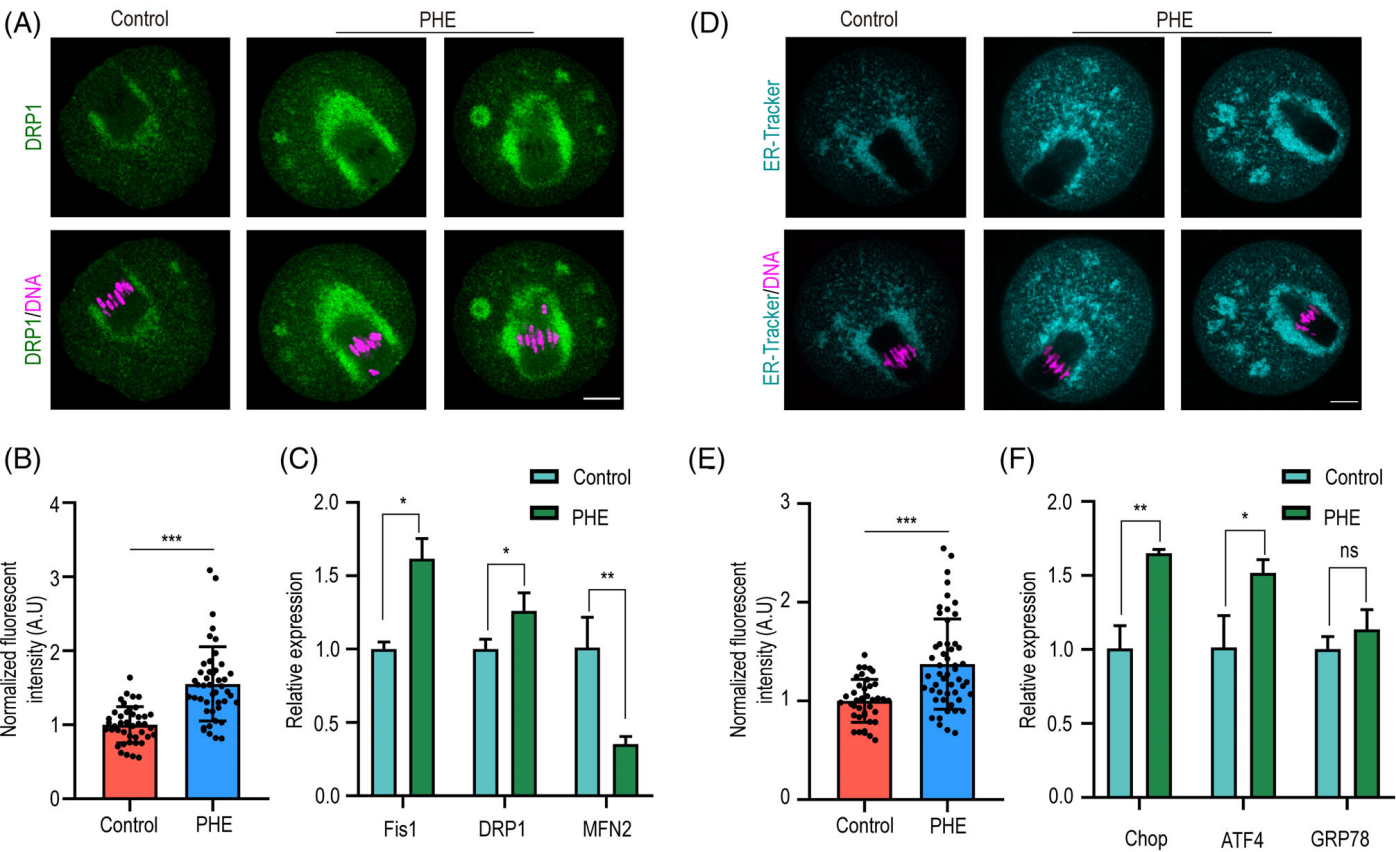
PHE exposure causes aberrant mitochondrial dynamics and endoplasmic reticulum stress. (A) Representative images of DRP1 in control and PHE ‐treated groups. Scale bar, 20 μm. (B) Normalized fluorescent intensity of DRP1 in control and PHE‐treated groups. (C) The relative mRNA expression of *Fis1*, *DRP1* and *MFN2* compared with the control group. (D) Representative images of ER‐tracker in control and PHE‐treated groups. Scale bar, 20 μm. (E) Normalized fluorescent intensity of ER‐tracker in control and PHE‐treated groups. (F) The relative mRNA expression of *Chop*, *ATF4* and *GRP78* compared with the control group. Each point in the histogram represents the number of oocytes. Data are represented as mean ± SD from at least three independent experiments. ns *p* > 0.05, *p < 0.05, **p < 0.01, ***p < 0.001

Given the importance of mitochondrial dynamics such as mitochondria fission and fusion during oocyte maturation, we investigated the effect of PHE on the expression level of Drp1, which played critical roles in mitochondrial fission. The fluorescent signal and statistical analysis results showed that the protein level of Drp1 was significantly increased after PHE exposure (1.0 ± 0.2, *n* = 42 versus 1.6 ± 0.5, *n* = 45, *p <* 0.001; Figure 4A,B). To further study the impact of PHE on mitochondrial dynamics, the changes in the mRNA expression of the related genes *Fis1*, *Drp1* and *Mfn2* were determined. The results showed that the mRNA levels of *Fis1* and *Drp1* were significantly increased, while *MFN2* was decreased (Figure 4C). It is well known that ER dysfunction always induces aberrant calcium homeostasis. Therefore, we next examined the amount of ER by ER‐tracker after PHE exposure. The result showed that PHE exposure caused a significant increase in fluorescence intensity of ER (1.0 ± 0.2, *n* = 39 versus 1.4 ± 0.5, *n* = 51, *p <* 0.001; Figure 4D,E). Meanwhile, the mRNA expression levels of ER stress‐relative genes, were measured by qPCR, such as *GRP78*, *ATF4* and *Chop*. Expectedly, the mRNA expression of *Chop* and *ATF4* were significantly increased, while the expression of *GRP78* was increased without significant difference (Figure 4F).

### 3.5 PHE induces ROS generation and DNA damage in oocytes

**FIGURE 5:**
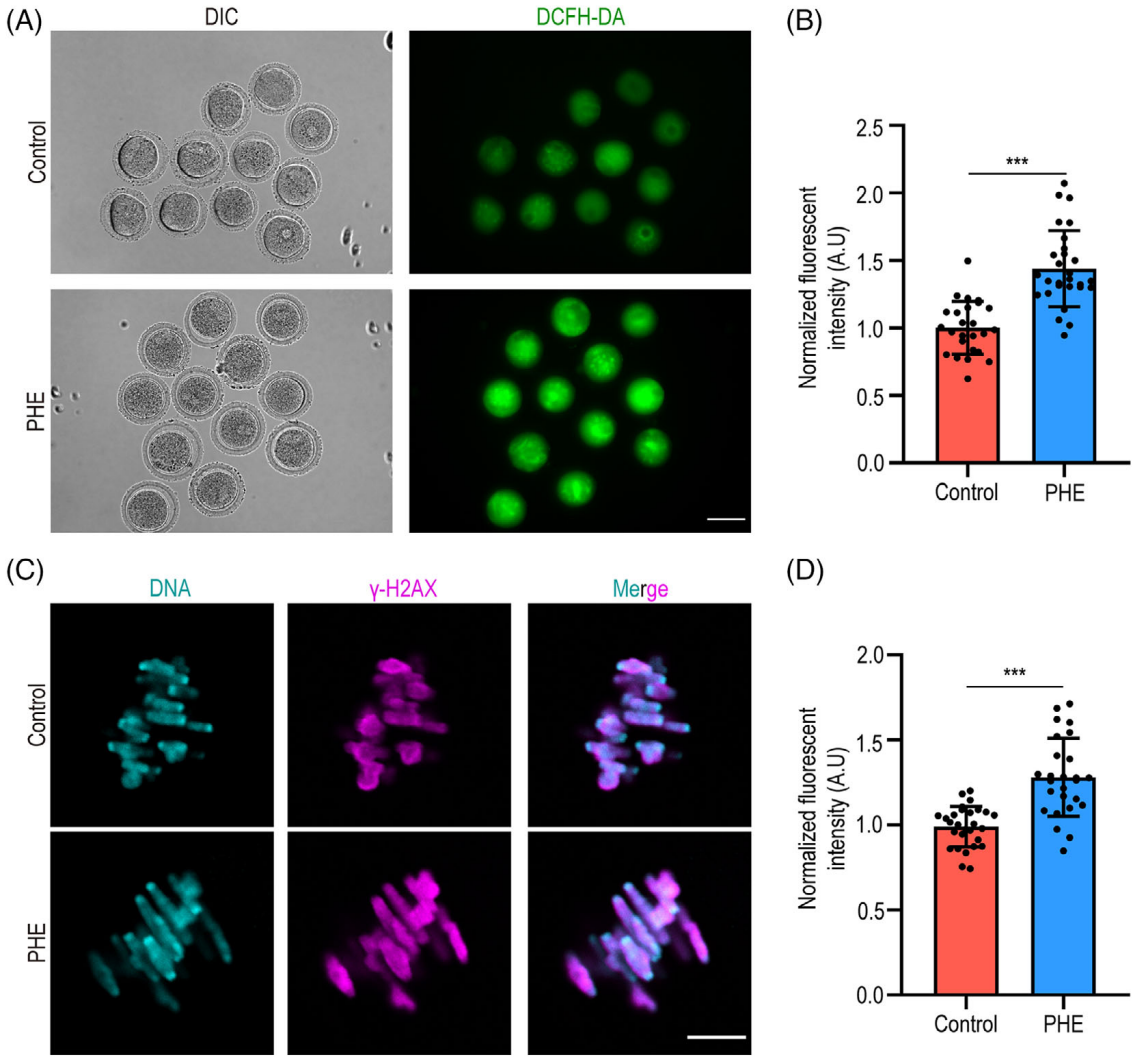
PHE exposure induces oxidative stress and DNA damage. (A) Representative images of DCFH‐DA staining in control and PHE‐treated groups. Scale bar, 100 μm. (B) Fluorescent intensity of ROS was analysed in control and PHE‐exposed oocytes. (C) Representative images of γH2AX staining in control and PHE‐treated groups. Scale bar, 10 μm. (D) Fluorescent intensify of γH2AX was quantified in control and PHE‐treated groups. Each point in the histogram represents the number of oocytes. Data are represented as mean ± SD from at least three independent experiments. ***p < 0.001

ROS were generated and consumed by mitochondria, while mitochondrial dysfunction usually induces oxidative stress accompanied by the accumulation of ROS, which has negative effect on oocyte quality. Accordingly, 2′‐7′dichlorofluorescin diacetate staining was used to measure the levels of ROS in oocytes. Expectedly, fluorescence intensity was significantly increased in PHE‐exposed group compared to controls, indicating that PHE exposure led to accumulation of ROS in oocytes (1.0 ± 0.2, *n* = 22 versus 1.5 ± 0.3, *n* = 25, *p <* 0.001; Figure 5A,B).

Given that excessive ROS can induce DNA damage, we next examined whether PHE exposure induced DNA damage during oocyte maturation by staining γH2AX. As shown in Figure 5C,D, the γH2AX signal was significantly increased after PHE treatment (1.0 ± 0.1, *n* = 28 versus 1.3 ± 0.2, *n* = 26, *p* < 0.001).

### 3.6 PHE causes oxidative stress and apoptosis in ovarian tissues and disturbs MII oocytes quality

**FIGURE 6:**
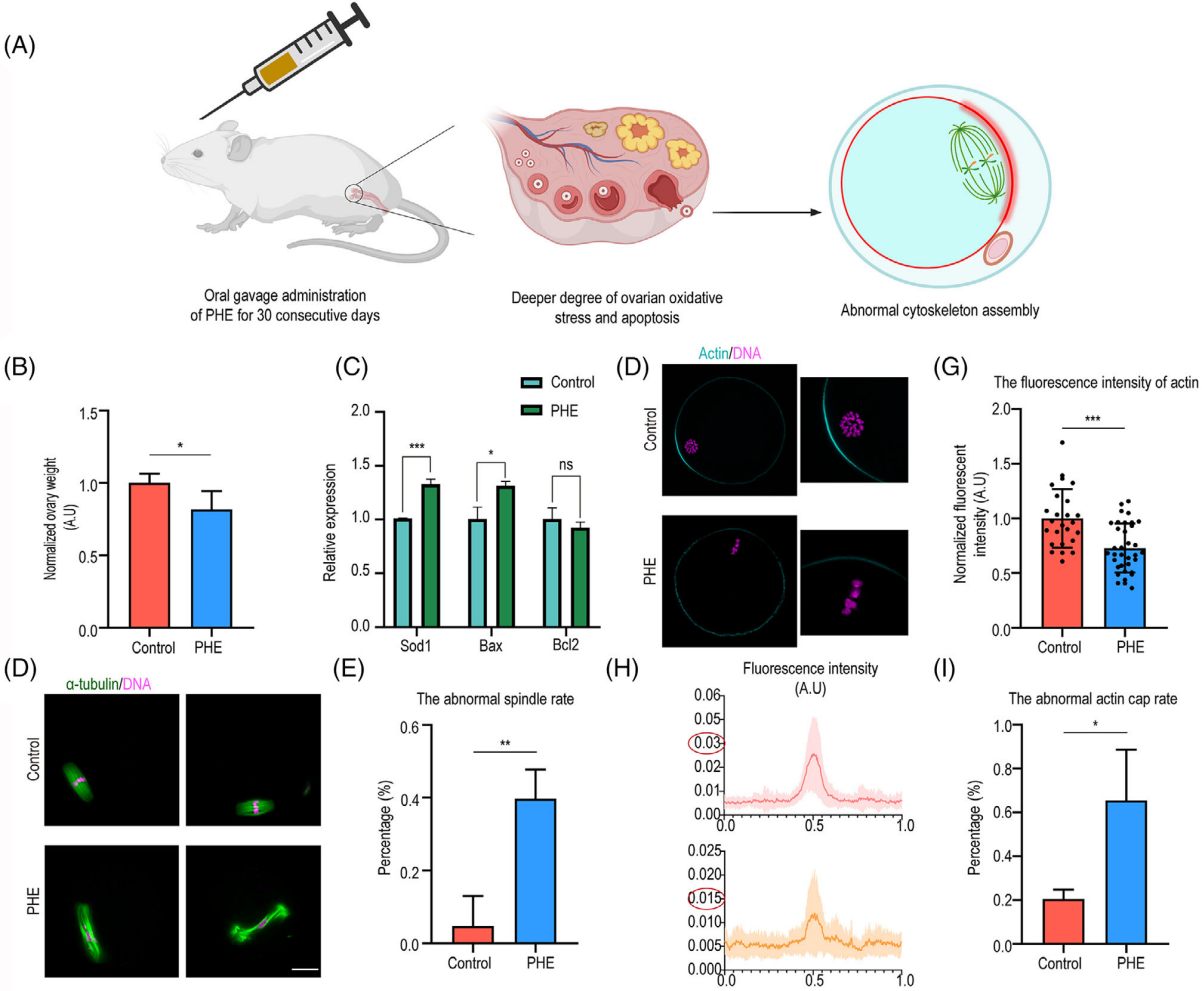
PHE causes oxidative stress and apoptosis in ovarian tissues and disturbs MII oocytes quality. (A) Schematic of the oral gavage administration of PHE and the effects on the ovaries. (B) Weight of ovarian in control and PHE‐treated groups. (C) The relative mRNA expression of *Sod 1*, *Bax* and *Bcl 2* in control and PHE‐treated groups. (D) Representative images of spindle morphologies in control and PHE‐treatment oocytes. Scale bar, 20 μm. (E) The rate of aberrant spindle was recorded in control and PHE‐treatment oocytes. (F) Representative images of actin on the cortical and the fluorescence intensity curves in control and treatment oocytes. Scale bar, 20 μm. (G) Fluorescent intensity of cortical actin was quantified in control and PHE treatment oocytes. (H) Cortical area actin fluorescence intensity curve was recorded in control and PHE‐treatment oocytes. (I) The rate of abnormal formation of actin cap was quantified in control and PHE treatment oocytes. Each point in the histogram represents the number of oocytes. Data are represented as mean ± SD from at least three independent experiments. ns *p* > 0.05, *p < 0.05, **p < 0.01, ***p < 0.001

To better understand the adverse effects of PHE on the female reproductive system, in vivo PHE exposure mice model was built by oral gavage administration for 30 days (Figure 6A). We found that the ovarian weights of PHE treatment mice were significantly reduced (1.0 ± 0.1, *n* = 4 versus 0.8 ± 0.1, *n* = 4, *p <* 0.05; Figure 6B). To further explore the effect of PHE on the ovarian function, the mRNA expression levels of antioxidase gene *Sod 1*, apoptosis‐related genes, *Bax* and *Bcl 2* were detected. The result showed that mRNA level of *Sod 1* and apoptotic gene *Bax* were markedly increased in PHE‐treated group (Figure 6C). These findings suggested that PHE exposure also induced oxidative stress and apoptosis in ovarian cells.

Subsequent to the first polar body extrusion and upon fertilization, the spindle of metaphase II was formed and played a critical role for fertilization. Therefore, we next investigated the effect of PHE exposure on cytoskeletal organization in MII oocytes. MII oocytes were obtained from the ampulla of the oviduct in PHE‐exposed mice. We observed that the percentage of aberrant spindle was remarkably increased after PHE exposure (4.8% ± 6.7%, *n* = 50 versus 39.7% ± 6.7%, *n* = 50, *p* < 0.01; Figure 6D,E). Next, we determined the effect of PHE on cortical polarization after PHE treatment by staining with phalloidin‐TRITC. As shown in Figure 6F,G, the fluorescence intensity of cortical actin was remarkably reduced after PHE‐treatment (1.0 ± 0.3, *n* = 25, versus 0.7 ± 0.3, *n* = 35, *p* < 0.001). Meanwhile, the actin cap was disappeared in most of PHE‐treated oocytes compared with that of control group (21% ± 3.4%, *n* = 40, versus 65.4% ± 3.4%, *n* = 45, *p <* 0.05; Figure 6H,I).

## 4 DISCUSSION

PAHs generated from organic combustion in industrial production^8^, ^12^, ^30^, ^31^ have been concerned by their estrogenic effects^32^ and potential harm to human,^33^ particularly the female reproduction system.^11^, ^34^ PHE is one of the most pervasive members of PAHs. As with other PAHs, various studies have shown that PHE exposure can cause reproductive damage. Recent studies showed that PHE exposure impaired spermatogenesis in male mice. In addition, PHE exposure inhibited ovary development and influenced the health of the offspring.^20^, ^35^ Gestational exposure of PHE reduced levels of luteinizing hormone and estradiol, as well as upregulation of the receptor of follicle‐stimulating hormone and aromatase in the ovary of offspring females. However, it is still unknown whether PHE has negative impacts on oocyte quality. In this work, we investigated the effects of PHE exposure on oocyte quality and ovarian function.

We first assessed the effect of PHE on oocyte maturation capacity in vitro. From the resumption of meiosis to maturation, the oocyte must undergo two landmark events: GVBD and PBE.^36^ The result showed that PHE exposure led to the failure of GVBD and PBE, suggesting that PHE significantly affected oocyte maturation. The spindle was a crucial organelle in oocyte meiosis, and aberrant spindle frequently resulted in the failure of meiotic processes. It has been shown that TPX2 was necessary for spindle assembly; aberrant TPX2 expression usually disrupted spindle morphology.^37^ Our results found that PHE exposure disrupted spindle assembly, accompanied by the increased expression of TPX2, indicating that PHE could induce abnormal microtubule assembly, and recruit more TPX2 to spindle for stabilizing spindle structure. During the first meiosis, microtubules and kinetochores interacted to drive chromosome alignment and segregation. Aberrant spindle morphology usually associated with chromosome misalignment and aneuploidy in oocytes.^38^, ^39^ As expected, measurement of chromosomes‐spread distance showed that most chromosomes were misaligned after PHE treatment.

During oocyte maturation, cortical polarization was crucial event for spindle asymmetric positioning and asymmetric cell division. It has been well documented that small GTPase superfamily played crucial role in cell polarization establishment.^40^, ^41^ Mutations or knockdown of Cdc42, a key member of the GTPase superfamily, led to abnormal actin assembly and failure of cell polarity establishment, which in turn blocked spindle migration and asymmetric cell division.^42^, ^43^ Meanwhile, Actin nucleation factor Arp2/3 complex were also indispensable in microfilament nucleation and cortical polarization establishment.^44^ Inhibition of Arp2/3 activity by CK666 blocked the formation of actin in MII oocyte and also caused rapid dissociation of spindle from cortex.^45^, ^46^ In the present study, we observed that the fluorescence intensity of actin in PHE exposure group was noticeably decreased than controls, and the actin cap was inconspicuous or disappeared. Meanwhile, we found that the frequency of abnormal MII spindle was noticeably increased after PHE exposure, indicating that PHE exposure could both inhibit the function of TPX2 and Arp2/3 complex in MII oocytes.

Many studies on PAHs exposure suggested that the toxicants impaired mitochondrial function.^38^, ^47^, ^48^ To adapt intracellular energy metabolism and redox homeostasis, mitochondria required continual fusion and fission throughout oocyte maturation.^49^, ^50^ In Drp1‐depleted oocytes, mitochondria were aggregated with other organelles, like ER, which disrupted the homeostasis of Ca^2+^ and oocyte quality.^51^ In addition, deletion of Mfn2 induced mitochondrial dysfunction, accompanied by the accumulation of ROS and increased apoptosis in Mfn2‐lacking oocytes.^52^ We found that, in this study, PHE treatment remarkably disrupted the balance of mitochondrial fusion and fission by inducing aberrant expression of *Drp1* and *Mfn2*. Imbalance of mitochondrial fission and fusion usually accompanied by the occurrence of oxidative stress, disrupting in calcium homeostasis and mitochondrial membrane potential.^53^, ^54^, ^55^, ^56^, ^57^ Consistent with this, we observed that PHE exposure remarkably increased ROS levels and intracytoplasmic Ca^2+^ in PHE‐exposed oocytes as well as decreased mitochondrial membrane potential in oocytes. Excessive accumulation of ROS in oocytes always led to DNA damage,^58^, ^59^ in line with this, we found that the level of DNA damage in PHE‐exposed oocyte was significantly higher than controls.

ER was the major intracellular organelle for protein synthesis. Dysfunction of ER induced the activation of UPR signalling pathways and ER stress, eventually blocked oocyte maturation.^29^ Moreover, ER was the major intracellular Ca^2+^ storage compartment, and disturbances in intracellular calcium homeostasis could induce dysfunction of ER.^60^ Our result showed that PHE treatment had no effect on ER distribution, but the amount of ER was significantly increased. Meanwhile, the expression of *Chop* and *ATF4* were significantly elevated. This may be caused by the massive enrichment of unfolded proteins in ER, which activated UPR signalling pathways and ER stress. Accordingly, our results indicated that PHE exposure could cause oxidative stress by inducing mitochondrial dysfunction and ER stress during oocyte maturation.

It has been abundantly demonstrated that PHE exposure led to increase of apoptosis in cells and tissues by increasing the activity of *caspase 3* and *caspase 9* enzymes and *Bax* expression.^14^, ^18^ And the apoptosis is most likely due to oxidative stress in tissues caused by PHE.^15^, ^61^ In line with this, our result demonstrated that PHE induced oxidative stress and apoptosis in ovary, which may explain the low quality of oocyte after exposure of PHE in vivo.

## 5 CONCLUSION

**FIGURE 7:**
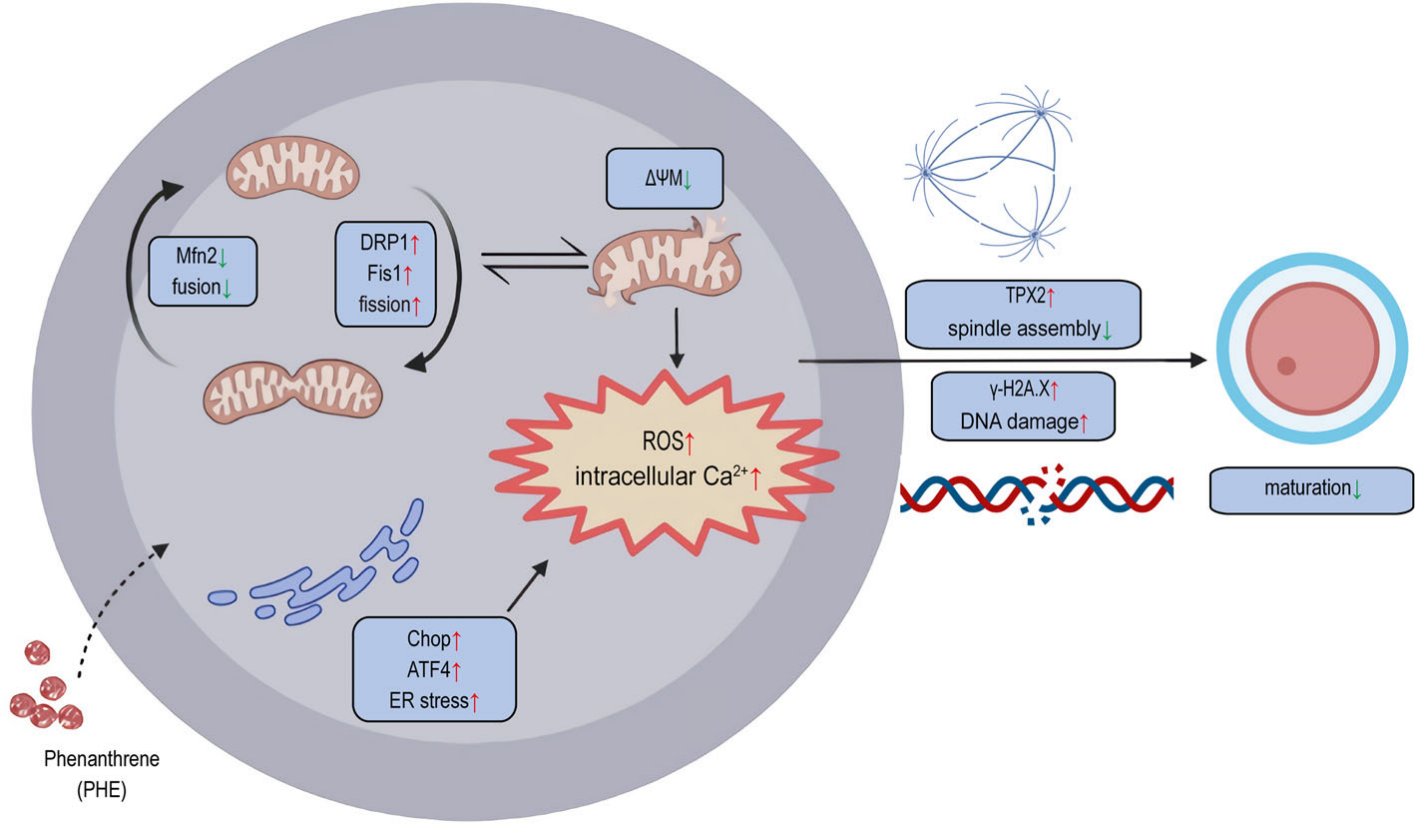
Mechanism of abnormal meiotic progression caused by PHE exposure. PHE exposure caused mitochondrial dysfunction by inducing the imbalance of mitochondrial dynamics and ER stress, which further resulted in the occurrence of DNA damage and reduced oocyte quality

Taken together, the current study indicated that PHE exposure caused ER stress and mitochondrial dysfunction by disrupting intracellular redox and Ca^2+^ homeostasis, eventually inducing DNA damage and apoptosis in oocytes (Figure 7). These findings expanded our knowledge of the influence of PHE on female reproductive capacity in mammals.

## AUTHOR CONTRIBUTIONS

Xing Duan designed the experiments and supervised the study. Yi Wang performed all experiments and analysed the data. Xiao‐Qing Li, Shu‐Jie Yang, Xiang Ma, Lu Liu and Dong Niu contributed to image analysis and discussion. Yi Wang, Si‐Hong Li and Xing Duan prepared the manuscript. All authors approved the submitted version.

## CONFLICT OF INTEREST

The authors declare that they have no known competing financial interests or personal relationships that could have appeared to influence the work reported in this article.

## Data Availability

All data generated or analyzed during this study are included in this published article.
